# Device‐Induced Blood Damage in Pump‐Assisted Circulation: A Comparative Study of HeartMate III and BrioVAD Pumps

**DOI:** 10.1111/aor.70166

**Published:** 2026-05-12

**Authors:** Wenji Sun, Kiersten P. Clark, Douglas Tran, John C. Vandenberge, Nancy Kim, Shigang Wang, Anik Tarafder, Randy Perez, Bartley P. Griffith, Dong Han

**Affiliations:** ^1^ Department of Surgery University of Maryland School of Medicine Baltimore Maryland USA

**Keywords:** blood damage, BrioVAD, HM3, in vitro, neutrophil, platelet, VADs, VWF

## Abstract

**Background:**

Mechanical circulatory support (MCS) has emerged as a life‐saving intervention and may be a potential alternative to organ transplant for patients with end‐stage heart failure. The Heartmate III (HM3) left ventricular assist device (LVAD) is a magnetically levitated centrifugal pump and the most advanced LVAD on the current MCS market. The BrioVAD is a newly developed LVAD featuring a fully magnetically suspended blood pump and has recently been approved by the FDA for the INNOVATE trial in the US. However, device‐related complications, which are highly related to blood damage by the pump, remain a major concern for patients with MCS. This study aimed to compare device‐induced blood damage by the HM3 and BrioVAD pumps in vitro in a circulatory loop.

**Methods:**

The device‐assisted circulatory loop was filled with fresh, healthy human blood. Both pumps were operated under the same clinically relevant condition, i.e., a blood flow rate of 4.5 L/min and an afterload pressure head of 75 mmHg, for 4 h. Hourly blood samples were collected to assess device‐induced damage to blood components, including red blood cells, platelets, neutrophils, and von Willebrand factor (VWF).

**Results:**

Both pumps had comparable impacts on blood components, with no statistically significant difference between them in all the measured parameters (except one neutrophil activation marker). After the four hours of device‐assisted circulation, both pumps caused minimal hemolysis and moderate platelet damage but had significant impacts on VWF and neutrophils.

**Conclusions:**

This study provides a comprehensive comparative in vitro analysis of blood damage caused by the HM3 and BrioVAD pumps under a clinically relevant condition. The results provide valuable insight into the potential implications for clinical use of the two devices and guidance for the design of new LVADs.

## Graphical abstract

Shear‐induced blood damage—including hemolysis, platelet and neutrophil injury, and von Willebrand factor (vWF) degradation—was evaluated for the BrioVAD and HM3 using an in vitro circulatory loop under clinically relevant conditions. Across 15 biomarkers (*n* > 6 per group), no significant differences were observed between the two devices.

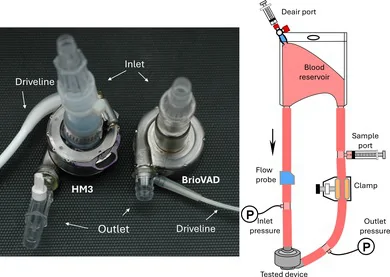

**Abbreviations**
GP: glycoprotein
HF: heart failure
HM2: HeartMate II
HM3: HeartMate III
HMWM: high molecular weight multimers
LVAD: left ventricular assist device
MCS: mechanical circulatory support
MFI: mean fluorescence intensity
NIH: normalized index of hemolysis
PBS: phosphate‐buffered saline
PFH: plasma‐free hemoglobin
PMPs: platelet‐derived microparticles
rpm: revolutions per minute
TMRE: Tetramethylrhodamine ethyl ester
VWF: von Willebrand

## 1 Introduction

Heart failure (HF) afflicts 6.7 million adults in the US and remains a critical health challenge. More than half of patients diagnosed with HF die within five years [1], and half of advanced HF patients (NYHA Class III and IV) die within one year [2]. While heart transplantation remains the only established effective treatment for advanced HF, the severely limited number of heart donors significantly restricts the number of transplants that can be performed, making heart transplantation an impractical universal therapy. Mechanical circulatory support (MCS), such as with left ventricular assist devices (LVADs), has emerged to offer a promising alternative therapy [3, 4, 5]. These devices mechanically pump blood, thereby replacing the heart's pumping function and maintaining blood circulation in patients with advanced HF, either as a bridge to transplantation or as destination therapy.

Since the discontinuation of the HVAD (Medtronic, Minneapolis, MN, USA) in June 2021, the HeartMate III (HM3) (Abbott Laboratories, Chicago, IL, USA) is the sole LVAD clinically available on the US MCS market. The HM3 is an advanced centrifugal LVAD featuring a fully magnetically levitated rotor that minimizes friction and wear, contributing to its durability and efficacy [6, 7]. Studies have demonstrated that the HM3 offers superior performance, including enhanced survival rates, improved quality of life, and reduced incidence of adverse events [8, 9, 10, 11]. The BrioVAD System, developed by BrioHealth Solutions (Burlington, MA, USA), is a novel centrifugal‐flow LVAD distinguished by its unique fully magnetic levitation design that enables a distinctive blood flow path design, smaller pump size, and thinner percutaneous driveline. More than 350 patients as of November 2024 outside the US have been treated with a ventricular assist system that incorporates the pump of the BrioVAD System. High survival and low complication rates have been reported in a study of 50 consecutive patients in Fuwai Hospital, China, demonstrating its safety and efficiency in providing long‐term support for end‐stage HF patients [12]. With Investigational Device Exemption approval from the US FDA, the BrioVAD System is currently undergoing a clinical trial in the US, namely INNOVATE [13].

Although the survival rate of patients supported with current LVADs is approaching that of heart transplantation, complications remain the major obstacle to their wide clinical use [8, 10, 11, 12, 14]. Hemostatic dysfunctions, such as nonsurgical bleeding and thrombosis, are the most significant issues associated with these devices and have direct implications for patient survival and quality of life. Device‐associated infection is also a major concern as it plays a critical role in adverse events and LVAD‐related complications. The LVAD‐associated complications are highly related to shear‐induced damage to blood components, including red blood cells, platelets, neutrophils, and von Willebrand factor (VWF). Although clinical data and numerical analyses are available to some extent for investigating device‐associated biocompatibility of the HM3 and BrioVAD devices [11, 15, 16], there are no studies that directly compare the biocompatibility performance of both pumps in vitro.

In this work, we aim to fill this gap by performing a comparative study of device‐induced blood damage between the HM3 and BrioVAD pumps in an in vitro circulatory loop. Using blood from healthy human donors, we evaluated both devices over a four‐hour period under a clinically relevant condition, monitoring key indicators of blood damage, including hemolysis, platelet and neutrophil dysfunctions, and VWF degradation. With the growing adoption of durable LVAD therapy and prolonged support durations, these devices are becoming an increasingly common treatment for advanced HF. We hope the present in vitro investigation provides a translational reference for comparative device biocompatibility, which may inform both clinical decision‐making and future device optimization strategies.

## 2 Materials and Methods

### 2.1 Device Description

**FIGURE 1:**
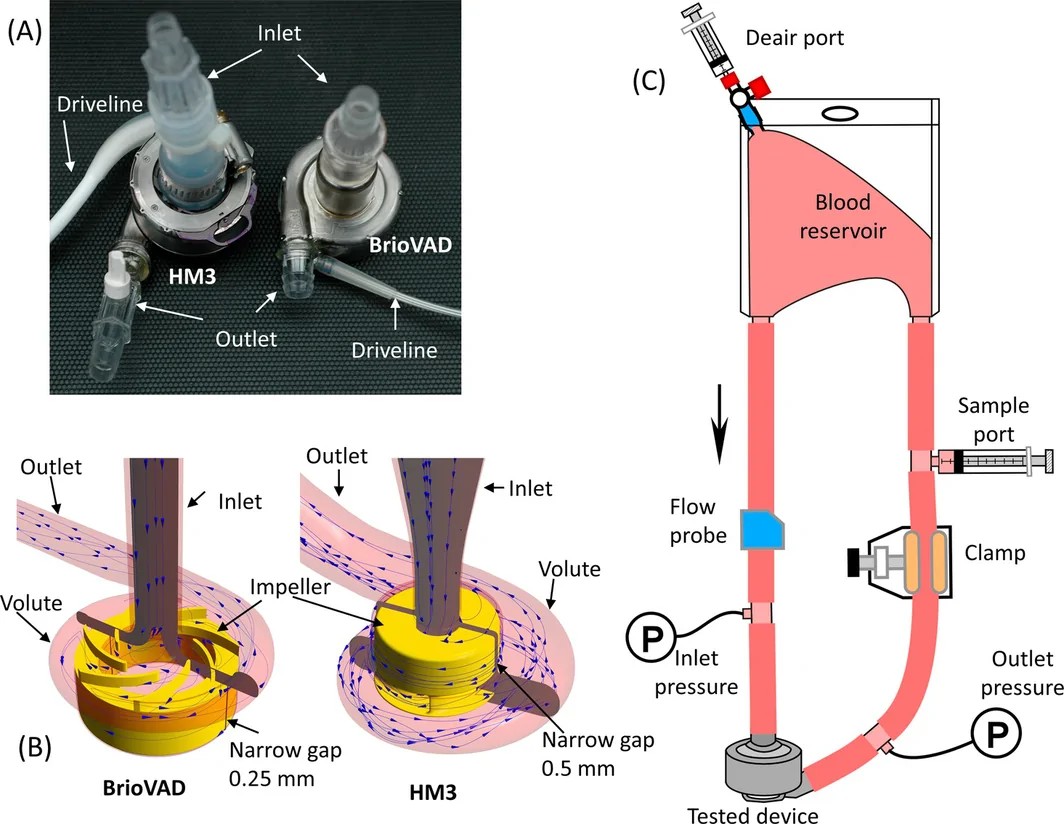
Overview of devices and experimental circuit setup. (A) Actual devices used in the study, (B) Characteristics of blood flow paths in the two devices, and (C) Schematic of the in vitro circuit setup. [Color figure can be viewed at wileyonlinelibrary.com]

The physical devices and blood flow paths of the HM3 and BrioVAD pumps are shown in Figure 1A,B. The HM3 features a fully magnetically levitated rotor, with a wide blood flow clearance of 0.5 mm along the sides, 1.0 mm above, and 1.75 mm below the rotor. These wide blood flow gaps are intended to minimize shear stress, reducing detrimental effects on blood components. The HM3 has an intrinsic artificial‐pulse mode to prevent stasis within the pump; the rotor changes speed every 2 s to generate pulsatile flow. The pump body has an outer diameter of 50 mm and a height of 40 mm. It has an impeller of 18.6 mm in diameter and operates at rotor speeds ranging from 3 000 to 9 000 rpm (rpm), achieving a maximum flow rate of 10 L/min. The HM3 weighs 200 g and has a prime volume of 15 mL.

The BrioVAD pump also has a fully magnetically levitated impeller, featuring a conical central strut that directs the axial primary flow into the impeller blade channels, preventing direct mixing of primary and secondary flows. It has a U‐shaped secondary flow path with a gap width of 0.25 mm, which compensates for sufficient flow washout, minimizes exposure time to high shear stress, and reduces flow turbulence to avoid Taylor vortices. The BrioVAD pump operates at a constant rotational speed without intended speed pulsation to manipulate flow inside the pump. The impeller has a 32.5 mm diameter and operates at speeds ranging from 1 000 to 4 200 rpm, providing a flow rate up to 10 L/min. The pump has an outer diameter of 47 mm and a height of 33 mm. It weighs 177 g with a prime volume of 9 mL.

### 2.2 Blood Collection and Circulatory Loop Setup

The blood donation was approved by the Institutional Review Board of the University of Maryland, Baltimore, and conducted in compliance with the Declaration of Helsinki. Blood was collected from healthy adult donors who had not taken antiplatelet or anticoagulant medications for two weeks prior to donation. All the participants were informed of the study's objectives and provided written informed consent. One unit of whole blood (450 mL) was drawn from the antecubital vein and stored in a sterilized blood bag (Teruflex dry collection bag, Terumo Corporation, Tokyo, Japan) containing 50 mL of 3.2% buffered sodium citrate solution (Medicago AB, Uppsala, Sweden) at room temperature. The hematocrit was adjusted to 35% ± 2% by hemodilution with phosphate‐buffered saline (PBS) with 0.5% bovine serum albumin, resulting in about 600 mL of blood [17].

Each experimental circulatory loop consists of a blood reservoir, a tested device, polyvinylchloride tubing (3/8‐in.), connectors, and ports for sampling or pressure measurements (Figure 1C). A parallel plate clamp, placed downstream of the blood pump, was used as a resistance throttle to adjust the pump pressure head. Before loading the blood into the two loops, a baseline blood sample was taken. Each circuit was then equally filled with approximately 300 mL of prepared blood. The experimental circuit was run at a low flow rate for approximately 5 min to mix the blood completely. By adjusting the rotating speed of the impeller and the resistance clamp, the pump was operated to generate a flow rate of 4.5 L/min and a pressure head of 75 mmHg at room temperature. The HM3 pump was operated at approximately 5 500 rpm, and the BrioVAD pump at 3 000 rpm using their authentic manufacturer‐supplied controllers. The HM3 was operated with its intrinsic artificial pulse mode, which transiently varied pump speed every 2 s, whereas the BrioVAD was operated at constant speed. The blood sample at hour zero was collected immediately after the circuit was established. Hourly blood samples were taken during the four‐hour experiment. More details about the in vitro loop setting can be found in our previous publications [18, 19].

### 2.3 Blood Sample Analysis

#### 2.3.1 Hemolysis Measurement

Hemolysis was assessed by measuring plasma‐free hemoglobin (PFH) concentration in hourly plasma samples using a spectrophotometric assay kit (Sigma‐Aldrich, St. Louis, MO, USA) with a SpectraMax M3 Spectrophotometer (Molecular Devices, San Jose, CA, USA). Then, the normalized index of hemolysis (NIH) was calculated according to the ASTM standard [17]. More details on hemolysis measurement can be found in the Supporting Information and in our previous publication [18].

#### 2.3.2 Flow Cytometry Analysis for Platelet Activation, Receptor Shedding, and Apoptosis

Platelet activation (P‐selectin and PAC‐1) and platelet surface adhesion receptors (glycoprotein (GP) Iba, GPVI, GPIIb/IIIa) shedding were measured by flow cytometric assays, as detailed in the Supporting Information and in our earlier publications [20, 21]. Briefly, whole blood samples containing 1 × 10^6^ platelets were added to the flow tube containing 50 μL of Tyrode's buffer (with 5 mM Gly‐Pro‐Arg‐Pro) and incubated with the antibody cocktails at room temperature in the dark for 20 min (a detailed summary of antibodies used in this study can be found in Table S1). The samples were then fixed with 1 mL of 1% paraformaldehyde in PBS for 30 min at 4°C. The flow data was acquired using a BD Calibur flow cytometer (BD Biosciences, San Jose, CA, USA) and analyzed with the FlowJo software (Ashland, OR, CA, USA).

Additionally, platelet‐derived microparticles (PMPs) in the blood samples were determined by the forward scatter characteristics of CD41a‐positive events, as a sign of platelet early activation. Annexin V was utilized to detect the externalization of phosphatidylserine, a critical marker of platelet apoptosis. For more details on these two measurements, refer to our previous publication [20]. Tetramethylrhodamine ethyl ester (TMRE) was assessed using the Mito PT TMRE assay (Immunochemistry Technologies, Bloomington, MN, USA) following the manufacturer's instructions. This assay was employed to evaluate mitochondrial function, providing additional insight into the state and activity of platelets' mitochondria, which is often indicative of platelet apoptosis.

#### 2.3.3 Flow Cytometry Analysis for Neutrophil Activation, Receptor Shedding, and Phagocytosis

Neutrophil activation and receptor shedding were assessed using flow cytometry as detailed in an earlier publication [22]. The expression of CD11b (Mac‐1), CD11a, and CD66b was evaluated for neutrophil activation, while receptor shedding was assessed by the surface expression of CD62L (L‐selectin). Briefly, 100 μL of whole blood was incubated at room temperature in the dark for 30 min with antibody cocktails. Corresponding isotype controls were prepared in the same manner. The cells were then fixed and lysed by adding 2 mL of eBioscience 1‐Step Fix/Lyse Solution (Invitrogen, Waltham, MA, USA) for 15 min. After two washes with flow cytometry PBS (FC‐PBS), the samples were acquired on a BD LSR II flow cytometer (BD Biosciences, San Jose, CA, USA) and analyzed using the FlowJo software.

Neutrophil phagocytosis was assessed using a phagocytosis assay kit (IgG‐DyLight 633, Cayman Chemical, Ann Arbor, MI, USA) following the manufacturer's instructions. In brief, 2 μL of DyLight 633 IgG‐coated beads was added to a 100 μL blood sample and incubated at 37°C for 60 min. The phagocytosis was stopped by placing the samples on ice for 5 min. Then the samples were stained with PE‐labeled anti‐CD66b Ab followed by 2 times washing with FC‐PBS. The neutrophils were identified by CD66b expression, and the percentage of DyLight 633 IgG‐positive cells among CD66b + was considered to represent neutrophil phagocytosis.

#### 2.3.4 Western Blotting Analysis of Multimeric VWF

The loss of the high molecular weight multimers of VWF (HMWM‐VWF) was assessed using Western blotting, following the procedure in our previous publications [21, 23]. Briefly, plasma samples were diluted 1:20 with Laemmli sample buffer (BIO‐RAD, Hercules, CA, USA) and separated on 0.6% SDS‐agarose gels. After transfer to a 0.45 μm nitrocellulose membrane (Thermo Fisher Scientific, Waltham, MA, USA), VWF multimers were detected using a polyclonal anti‐VWF antibody and an HRP‐conjugated secondary antibody. Visualization was performed on a BIO‐RAD ChemiDoc Imaging System, and band intensities were quantified with UN‐SCAN‐IT Gel 6.1 software (Silk Scientific, Orem, UT, USA). The HMWM proportion was defined as the ratio of HMWM‐VWF band pixel intensity to the total pixel intensity of all VWF bands. VWF degradation was subsequently quantified as the fold change in HMWM proportion relative to its baseline value.

#### 2.3.5 Data Analysis

The measurements for the above‐listed indicators for device‐induced blood damage in the circulatory loop were repeated at least 6 times. The data is presented as mean ± SE (standard error). GraphPad Prism software version 10.0.2 (GraphPad Software, La Jolla, CA, USA) was used for a Two‐way ANOVA (or mixed‐effects model) analysis followed by a Bonferroni multiple comparison test. The *p* values for device and time are summarized in Table 1. *p* values < 0.05 were considered significant for all statistical tests.

**TABLE 1:** Summary of P‐values for device type and experimental time.

Biomarker	*p*	
	Device	Time
P‐selectin	0.5588	0.0059
PAC‐1	0.9800	0.0030
GPIbα	0.8384	< 0.0001
GP VI	0.2117	0.0002
GP IIb/IIIa	0.7943	< 0.0001
Annexin V	0.1267	0.0026
MPMs	0.8876	< 0.0001
TMRE	0.1356	0.1964
CD11b (Mac‐1)	0.0225	< 0.0001
CD62L	0.3149	< 0.0001
CD11a	0.3149	< 0.0001
CD66b	0.2381	< 0.0001
Phagocytosis	0.8259	0.0197
HMWM VWF	0.5416	< 0.0001
NIH (*t*‐test)	0.8476	

## 3 Results

The calculated NIH value was 0.00063 ± 0.00022 g/100 L for the HM3 and 0.00068 ± 0.00014 g/100 L for the BrioVAD, respectively. There is no statistically significant difference between the HM3 and BrioVAD pumps. Additional data related to hemolysis, including PFH and the modified index of hemolysis (MIH), are provided in the Supporting Information. A summary of hematology data, including platelet count, white blood cell count, and hematocrit, is also provided.

**FIGURE 2:**
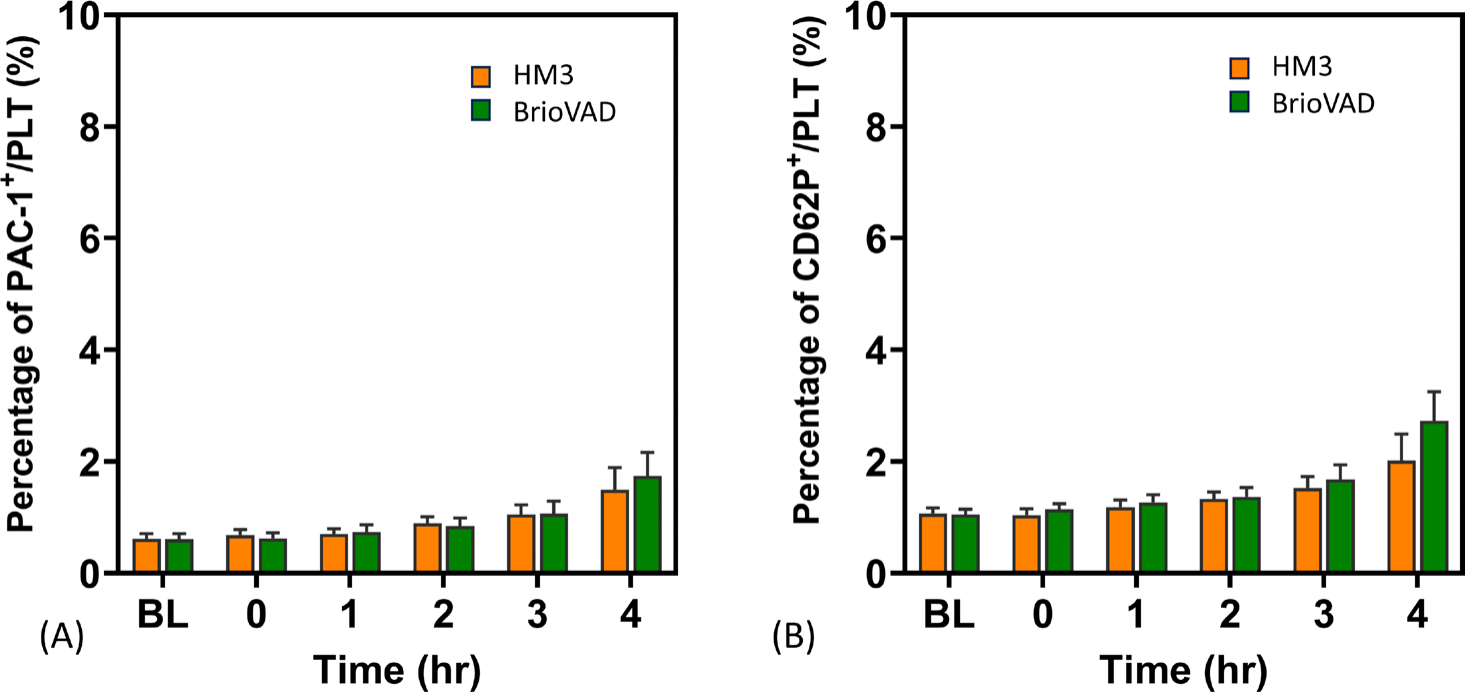
Platelet activation levels over time in both devices. (A) PAC‐1, and (B) P‐selectin (CD62P). [Color figure can be viewed at wileyonlinelibrary.com]

Figure 2 shows the changes in platelet activation levels, measured by P‐selectin and PAC‐1, over time in both loops from baseline. No statistically significant differences were observed between the two devices. Blood samples from both loops with the two devices exhibited a statistically significant increase in P‐selectin and PAC‐1 expression over time. However, the increases in platelet activation caused by both pumps were less than 3% even at hour four, suggesting excellent biocompatibility with respect to platelet activation in both devices.

**FIGURE 3:**
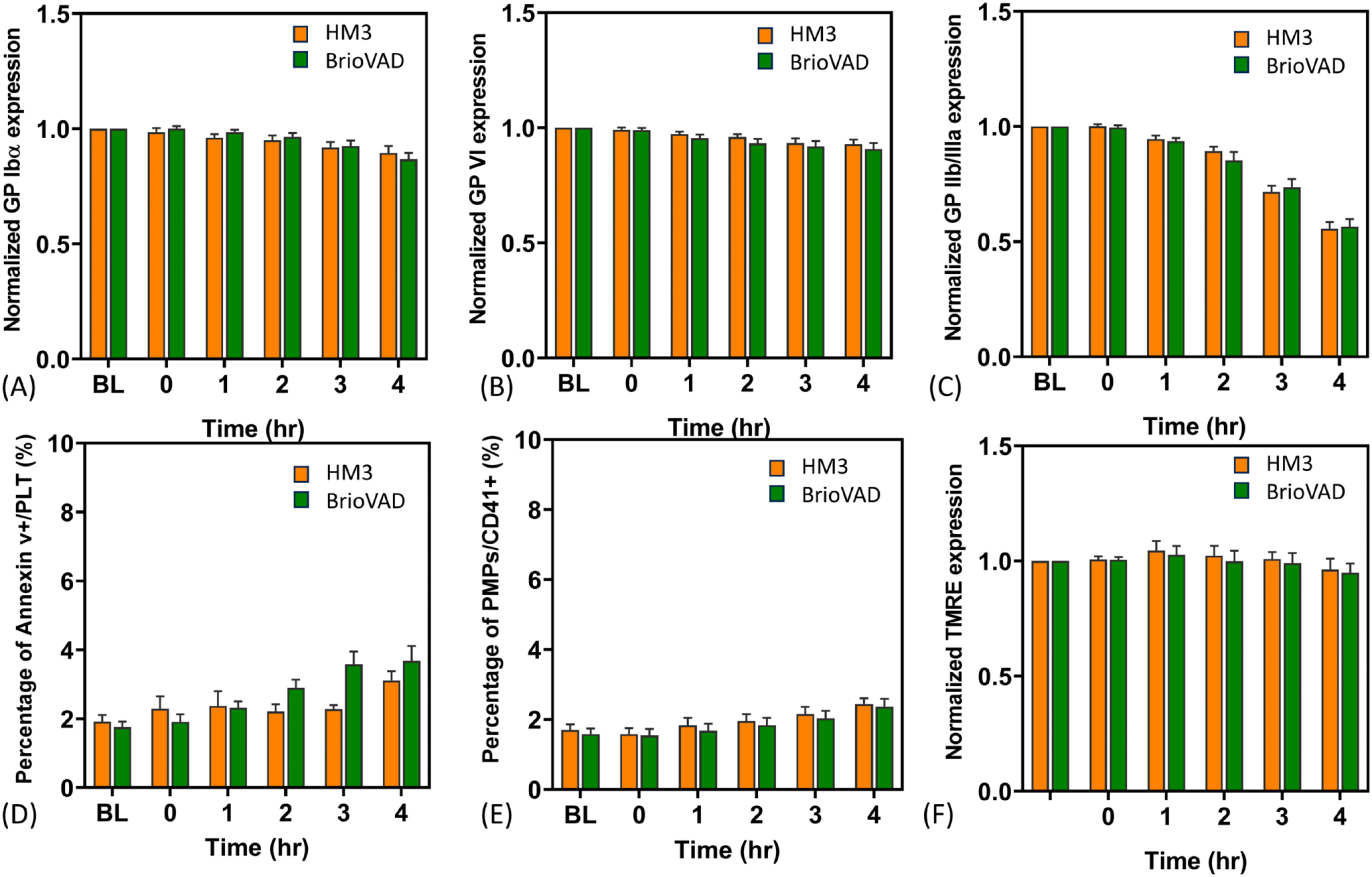
Measurement of platelet receptor shedding, apoptosis, and PMPs over time in both devices. Normalized receptor shedding of (A) GPIbα, (B) GPVI, and (C) GPIIb/IIIa relative to baseline blood samples. (D) Annexin V+ platelets, (E) PMPs, and (F) normalized platelet TMRE expression levels over time in both devices. [Color figure can be viewed at wileyonlinelibrary.com]

The changes in the mean fluorescence intensity (MFI) of three key platelet receptors (GPIbα, GPVI, and GPIIb/IIIa) in hourly blood samples from the loop with the HM3 or BrioBAD pump are illustrated in Figure 3A–C. The MFI values are normalized to baselines (see all baseline values in Table S2). The levels of three receptors decreased over time, indicating increased receptor shedding on platelets. The decreasing trends of the three surface receptors over time between the two pumps are almost identical. No significant differences between the two devices during the four‐hour circulation were found for GPIbα, GPVI, and GPIIb/IIIa. However, all three receptors experienced a significantly increased shedding over time. Compared to GPIbα and GPVI, GPIIb/IIIa had a greater loss, especially at hour four for both devices (~40%), indicating the susceptibility of this receptor to mechanical shear stress.

The measurements of Annexin V, platelet MPMs generation, and TMRE for platelets are shown in Figure 3D–F, respectively. A slight increase in the percentage of Annexin V^+^ platelets and PMPs was observed in hourly blood samples over time; however, the difference between the two devices was not statistically significant. The increases in Annexin V and PMPs were statistically significant over time. For TMRE, no significant differences over time or between the devices were observed.

**FIGURE 4:**
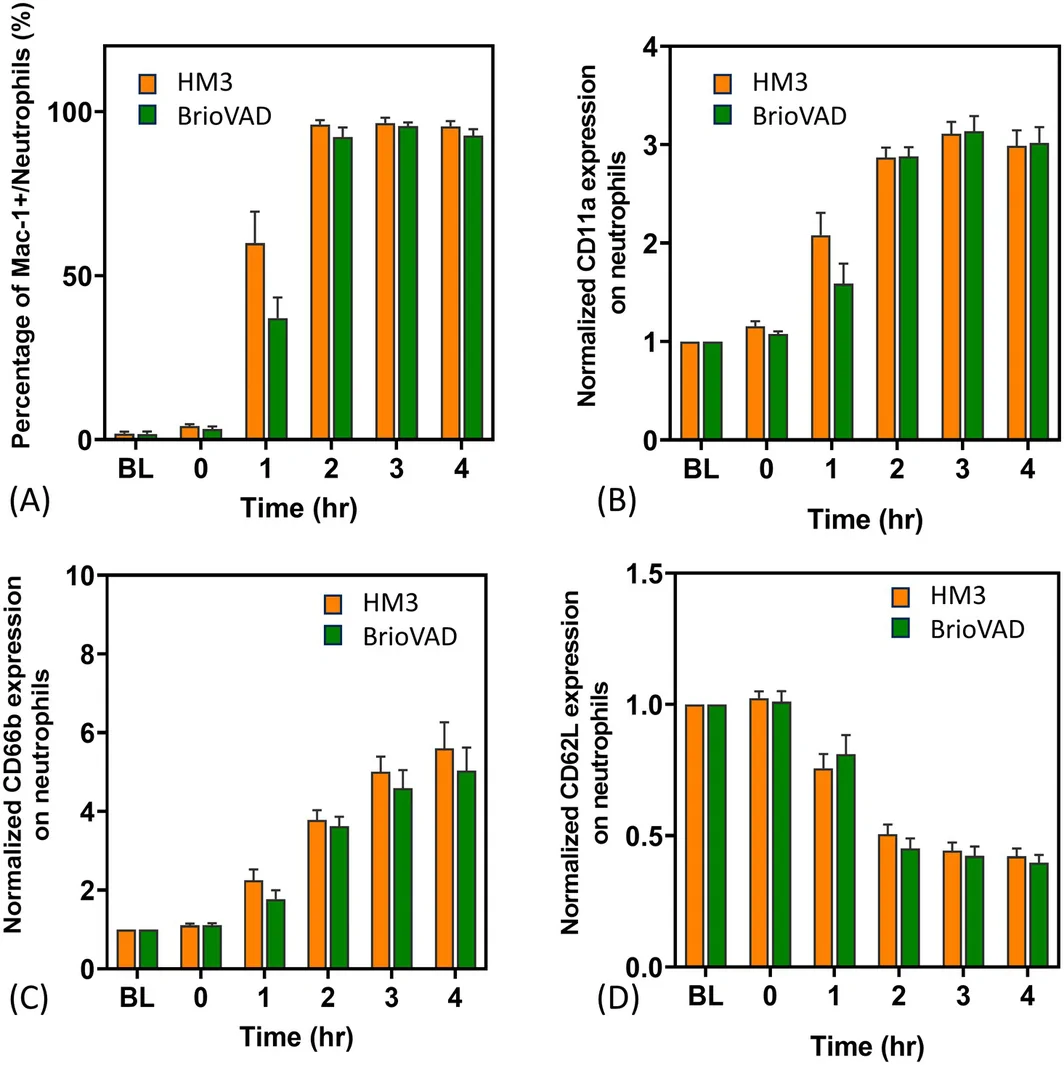
Neutrophil activation and CD62L receptor shedding in both devices over time. (A) Percentage of Mac‐1+ neutrophils, (B) Normalized CD11a expression, (C) Normalized CD66b expression, and (D) Normalized CD62L expression. [Color figure can be viewed at wileyonlinelibrary.com]

Neutrophil activation, as indicated by the upregulation of CD11b (Mac‐1), CD11a, and CD66b expression, and neutrophil receptor shedding, as indicated by the downregulation of CD62L expression, is shown in Figure 4. All neutrophils became activated after two hours of circulation in the loop for both devices, as indicated by the increase in percentage of CD11b^+^ population (Mac‐1%) to nearly 100% (Figure 4A). However, there was a significant difference between the HM3 and BrioVAD for Mac‐1%, with the HM3 showing a significantly higher level of Mac‐1% at hour one compared to the BrioVAD. In addition, CD11b and CD66b MFI expressions on neutrophils, normalized to baseline values, were significantly upregulated for both devices (Figure 4B,C). No statistically significant differences in these two activation biomarkers between the BrioVAD and HM3 devices were observed. The surface expression of CD62L significantly decreased with time, with the most pronounced reduction observed at hour four; the CD62L expression level decreased to 59% for the HM3 pump and 61% for the BrioVAD pump compared to the baseline level (Figure 4D). There was no significant difference in CD62L expression between the two devices.

**FIGURE 5:**
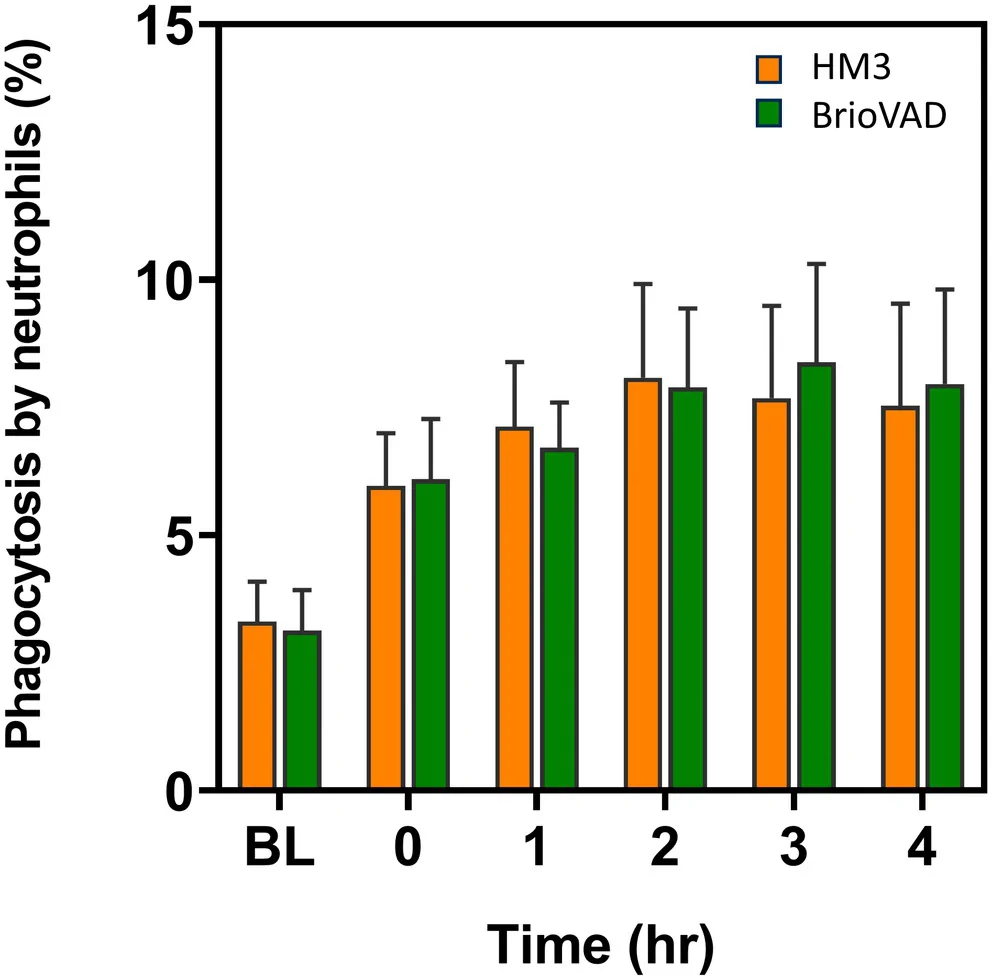
Percentage of CD66b + neutrophils, indicating phagocytic ability, from the blood samples in circulation with both devices. [Color figure can be viewed at wileyonlinelibrary.com]

Figure 5 shows the change in phagocytic ability of neutrophils over time for the HM3 and BrioVAD pumps. Neutrophil phagocytic activity had a marked increase at hour zero compared to baseline level for both devices, indicating that the intrinsic phagocytic potential was elicited after being subjected to device‐assisted circulation. No significant difference was observed between the devices. In the HM3, neutrophils' phagocytic ability peaked within the first two hours and then leveled off over the remaining hours. Similarly, in the BrioVAD, neutrophil phagocytic ability increased until hour three before gradually leveling until the final hour of circulation.

**FIGURE 6:**
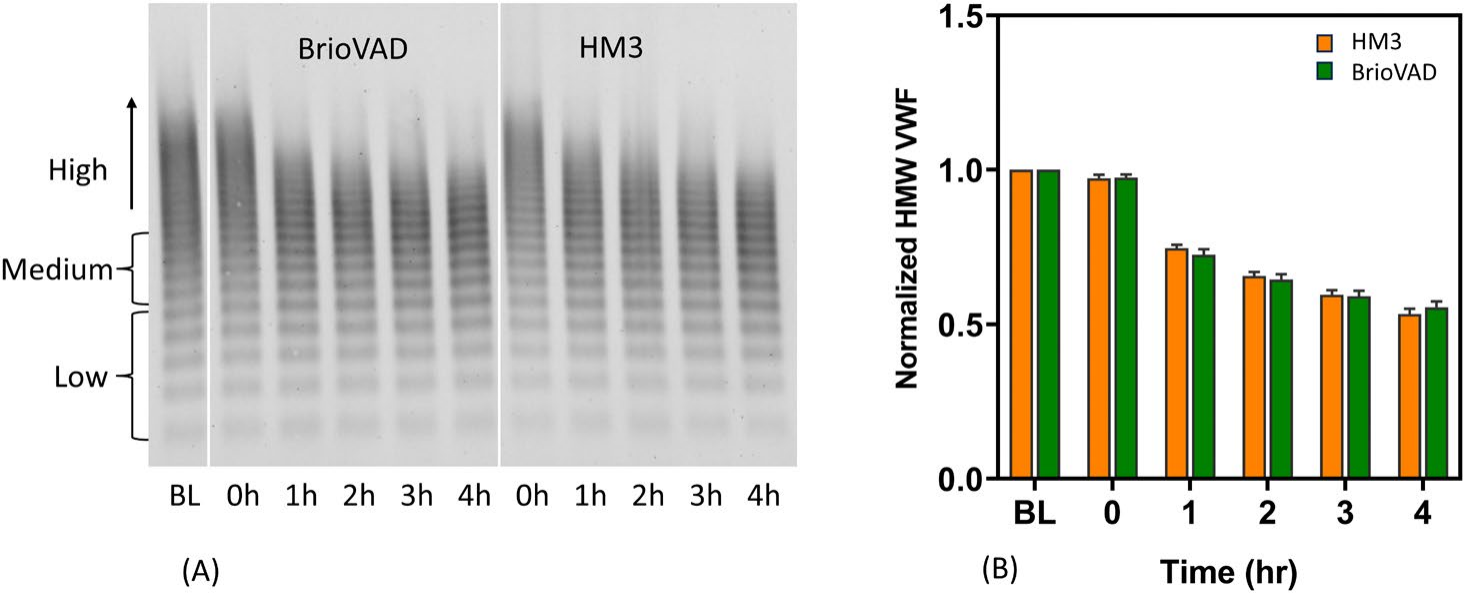
Analysis of VWF multimeric profiles. (A) Representative Western blot image showing the VWF multimeric profile from baseline and hourly samples during circulation for both devices. (B) Normalized HMWM‐VWF proportions over time for both devices. [Color figure can be viewed at wileyonlinelibrary.com]

Figure 6A shows representative Western blot images of the multimeric profiles of VWF at baseline and over four consecutive hours for the HM3 and BrioVAD pumps. Figure 6B quantifies the temporal fold changes in HMWM proportion for each device, normalized to baseline. The HMWM‐VWF proportion is almost identical between the two devices over time, with no statistically significant difference at any time point. However, there is a clear significant decrease in HMWM proportions over time, with about half of HMWM‐VWF degraded by hour four (53% remaining in the HM3 and 56% remaining in the BrioVAD, respectively).

## 4 Discussion

With advancements in technology, the current fully magnetically levitated LVADs have significantly increased the survival rates of patients with end‐stage HF. In a multicenter study involving nearly 300 patients, Mehra et al. compared the clinical outcomes between the HM3 centrifugal flow pump and the HeartMate II (HM2) (Abbott Laboratories, Chicago, IL, USA) axial flow pump in patients with advanced HF who did not respond to standard medical therapy [11]. The study showed that the HM3 achieved better overall outcomes, with fewer major postoperative complications, including reduced incidences of stroke and bleeding. Other studies also supported the indication for long‐term, permanent use [9, 10]. A study of an early version of the BrioVAD system in a cohort of 50 patients had shown that the survival rate of patients on BrioVAD support was high, with a low incidence of adverse events and hemocompatibility complications [12]. Despite the relatively younger patients that were enrolled, this single‐center study confirmed the safety and efficacy of the BrioVAD LVAD for short‐ and long‐term support of patients with end‐stage HF.

Although patients on the HM3 LVAD show reduced rates of stroke and bleeding compared to the HM2 LVAD, these complications still represent significant challenges in clinical practice. Infections—including sepsis, driveline infections, and other device‐unrelated infections—are more frequent in the HM3 than in the HM2. This trend underscores the critical need for addressing infection control as an integral aspect of device design and postoperative care. Similarly, infection remains the most common adverse event among BrioVAD patients.

It is well known that non‐physiological shear stress produced by the mechanical pumping action of LVADs can damage blood components [24], such as red blood cells, platelets, neutrophils, and VWF. Damage to these key components is closely linked to complications in patients. For example, damage to red blood cells, also known as hemolysis, leads to increased PFH, which can cause acute kidney injury [25]. Damage to platelets and VWF can cause platelet dysfunction and VWF degradation, both of which are strongly associated with hemostasis disorders, such as bleeding and thrombosis in LVAD patients [26]. Leukocyte damage can elevate proinflammatory cytokines, impair cellular immunity, and activate leukocytes, all of which are likely contributors to the inflammatory complications observed in patients following LVAD implantation [22, 27].

In this study, we conducted a comparative study between the HM3 and BrioVAD pumps in an in vitro circulatory loop, focusing on the biocompatibility in terms of the blood damage associated with the two devices. Although the blood flow designs inside these pumps are markedly different [12], this study found that both devices exhibited similar biocompatibility performance across all measured indicators. These results suggest that the two LVADs have comparable biocompatibility safety profiles. Both the HM3 and BrioVAD pumps caused minimal red blood cell damage as shown by NIH values, which are one order of magnitude lower than previously reported results for the old LVADs, such as HVAD and HM2 [18, 28]. Although hemolysis has historically been a critical index for the biocompatibility of blood pumps, advances in fully magnetically levitated pumps may have significantly reduced its clinical impact on red blood cells in patients.

Our results also suggest that both devices caused low platelet activation, with less than 3% increase in platelet activation at the four‐hour mark. Mild receptor shedding of GPIbα and GPVI was observed in the blood samples over time in the loop with either the HM3 or BrioVAD pump, while GPIIb/IIIa exhibited a markedly higher level of shedding over time in both devices. The changes in annexin V expression, PMPs generation, and TMRE were relatively low (~2%) during the four‐hour circulation. The overall biocompatibility profiles of device‐induced platelet damage between the two devices were quite similar. In contrast, both devices had a significant effect on VWF degradation over time, with reductions of 47% and 43% in HMWM‐VWF after four hours of circulation in the HM3 and BrioVAD groups, respectively.

Neutrophils are among the first‐line immune cells to respond to exotic pathogens, eliminating these alien invaders via rolling, adherence, transmigration, and phagocytosis [29, 30]. Both the HM3 and BrioVAD pumps had a significant effect on neutrophil activation, as evidenced by increased expression of the biomarkers Mac‐1, CD11a, and CD66b. The BrioVAD had slightly lower neutrophil activation compared to the HM3, although the difference was overall not statistically significant (except Mac‐1). Both devices caused a significant decrease in expression of the key neutrophil receptor CD62L, with a reduction of ~60% at hour four compared to the baseline level. Unlike other biomarkers, which remained relatively stable at hour zero compared to the baseline levels, neutrophil phagocytic activity exhibited a noticeable increase immediately after blood was loaded into the loops, underscoring its heightened susceptibility to the non‐physiological environments of the circulatory loop. Despite overall improvements in biocompatibility, particularly regarding hemolysis and platelet injury, changes in biomarkers for neutrophil activation, receptor shedding, and phagocytosis suggest that both pumps had significant effects on neutrophils over the four‐hour circulation. These changes could impair their function and contribute to infection risk due to abnormal preemptive neutrophilic migration and phagocytosis. This phenomenon may explain why infections, as observed in clinical studies, remained a major complication in LVAD patients using either device.

## 5 Limitation

This study used blood from healthy donors, which may not fully reflect the physiological conditions of patients with HF. The HM3 pump had been previously used clinically prior to explantation, whereas the BrioVAD pump was newly obtained. The BrioVAD and HM3 pumps were reused, although they were thoroughly cleaned each time. Contact with surfaces such as the reservoir bag and tubing in the circuit may increase the exposure of blood cells to foreign surfaces, particularly affecting neutrophils, which play a critical role in defense against and clearance of foreign substances. The blood volume in the circulatory loop was relatively small compared with that in the human body. The experiments were conducted at room temperature for 4 h. A single operating condition (4.5 L/min at 75 mmHg pressure head) was used, which may not fully represent the range of clinical scenarios for these devices. Therefore, caution should be exercised when extrapolating the results to the clinical setting.

## 6 Conclusion

The overall biocompatibility performance of the HM3 and BrioVAD pumps is similar across all assessed aspects in this in vitro loop study. Notably, our findings confirm that while improvements in device design have mitigated certain blood component damage, challenges remain, especially with damage to neutrophil and VWF, which may contribute to ongoing risks of infection and bleeding. Neutrophils, in particular, experience severe overall damage compared to platelets and red blood cells, which may help explain why infections are a major complication in VAD patients observed in patients. VWF also undergoes substantial damage, highlighting its critical role in hemostasis regulation. Thus, beyond comparing these two clinical LVADs, this study also provides valuable insights for future design improvements and therapeutic strategies for patients on MCS.

## Author Contributions

Wenji Sun: Study design, experiment execution, data collection and statistical analysis, manuscript writing and revision. Kiersten P. Clark: Experiment execution, data collection and analysis, manuscript revision. Douglas Tran: Experiment execution, manuscript revision. John Vandenberge: Experiment execution, manuscript revision. Nancy Kim: Experiment execution, manuscript revision. Shigang Wang: Study design, experiment execution, data collection and analysis. Anik Tarafder: Manuscript revision. Randy Perez: Experiment execution, data collection. Bartley P. Griffith: Study concept and design, manuscript review, funding acquisition. Dong Han: Study concept and design, data analysis and interpretation, article approval, manuscript writing and revision, funding acquisition.

## Funding

This work was supported by the National Institutes of Health (R01HL118372, R01HL162940, and R01HL171120).

## Disclosure

Research reported in this publication was supported by the National Heart, Lung, and Blood Institute of the National Institutes of Health under Award Nos. R01HL118372, R01HL162940, and R01HL171120. The content is solely the responsibility of the authors and does not necessarily represent the official views of the National Institutes of Health. B.P.G. discloses financial interest in Abiomed‐Breethe Inc. and intellectual property for ECMO systems, blood pumps, and relevant enabling technologies. All other authors declare that they have no conflict of interest in the subject matter or materials reported in this study.

## Conflicts of Interest

The authors declare no conflicts of interest.

## Supporting information


**Figure S1:** shows the change in plasma‐free hemoglobin (PFH) over time. For both devices, PFH increased slightly from a baseline of approximately 22 mg/dL to approximately 28 mg/dL over the duration of the experiment. The overall increase in PFH was relatively small; however, the trend remained approximately linear with respect to time. At this low level of PFH change, the signal approaches the sensitivity limits of the measurement, and therefore, a relatively larger proportion of the observed variation may be attributed to measurement uncertainty.
**Figure S1:** Change in PFH over time.
**Figure S2:** Calculated MIH.
**Figure S3–S5:** summarizes the hematology data, including platelet count, white blood cell (WBC) count, and hematocrit (HCT). These parameters were measured using an automated hematology analyzer, with hematocrit additionally verified by microcentrifugation. The results show that these parameters remained relatively stable over the 4‐h loop study, despite potential sublethal damage to some cells.
**Figure S3:** Platelet count.
**Figure S4:** WBC count.
**Figure S5:** HCT.
**Figure S6:** shows the analysis strategy for platelet activation and receptor shedding. Platelets were first identified by gating the CD41a+/SSC^low population. Activated platelets were defined as CD62P+ or PAC‐1+ events within the CD41a+ population. Receptor shedding was assessed by measuring the expression levels of GPIbα, GPVI, and GPIIb/IIIa within the CD41a+ population. Representative density plots are shown from a blood sample in the BrioVAD loop at hour 4. The flow cytometry gating strategy for neutrophils has been described in detail in the main manuscript and our previous publications [22].
**Figure S6:** The flow cytometry gating strategy for platelet activation and receptor shedding.

**Table S1:** List of monoclonal antibodies used in this study.
**Table S2:** Baseline values for all the biomarker measurements.

## Data Availability

The data that support the findings of this study are available from the corresponding author upon reasonable request.
