# Supplementary material for: Device‐Induced Blood Damage in Pump‐Assisted Circulation: A Comparative Study of HeartMate III and BrioVAD Pumps
Source: Artif Organs. 2026 May 12;50(9):1295–304. doi: 10.1111/aor.70166 (PMC13525217; doi:10.1111/aor.70166)
Supplement: Supplementary file 1 [file AOR-50-1295-s001.docx]

## Hemolysis

**Figure S1** shows the change in plasma free hemoglobin (PFH) over time. For both devices, PFH increased slightly from a baseline of approximately 22 mg/dL to approximately 28 mg/dL over the duration of the experiment. The overall increase in PFH was relatively small; however, the trend remained approximately linear with respect to time. At this low level of PFH change, the signal approaches the sensitivity limits of the measurement, and therefore a relatively larger proportion of the observed variation may be attributed to measurement uncertainty.

According to the ASTM F1841-97, the normalized index of hemolysis (NIH) was calculated using the following equation:

NIH = (ΔPFH × V × (100 − Hct)) / (100 × Q × T) × 100

where ΔPFH is the increase in plasma free hemoglobin (g/L), V is the blood volume in the loop (L), Hct is the hematocrit (%), Q is the flow rate (L/min), and T is the circulation time (min). NIH is expressed in units of g/100 L. Measured PFH values (mg/dL) were converted to g/L prior to calculation.

Because samples were collected hourly, ΔPFH/T was calculated based on the slope of the PFH versus time curve obtained from linear regression, rather than from any single time interval. The NIH values were then averaged across all experimental runs.

The calculated NIH values for both loops are reported in the main manuscript and were low, indicating minimal red blood cell damage under the tested conditions, consistent with the small increase in PFH observed over time. The modified index of hemolysis (MIH) was calculated based on the ASTM formulation:

MIH = ΔPFH × V × (100 − Hct) / (100 × Q × T × Hgb) × 10^6^

Note that in this equation, ΔPFH and Hgb (hemoglobin concentration) are expressed in mg/L. For convenience, MIH can also be expressed in terms of NIH as:

MIH = 1000 × NIH / Hgb

where NIH is in g/100 L and Hgb is in g/dL. The MIH bar chart is also reported in **Figure S2** to provide an additional reference for interpretation of hemolysis results.

PFH(mg/dL)

|  | BrioVAD | HM3 |
| --- | --- | --- |
| base | 21.0311 | 22.6887 |
| 0H | 19.9539 | 23.9627 |
| 1H | 25.3794 | 25.4647 |
| 2H | 24.296 | 24.9547 |
| 3H | 30.5704 | 28.0318 |
| 4H | 28.2618 | 28.1642 |

**Figure S1**. Change in PFH over time.

|  | BrioVAD | HM3 |
| --- | --- | --- |
| NIH | 0.0670928 | 0.0639057 |

**Figure S2**. Calculated MIH.

## Hematology

**Figure S3-5** summarizes the hematology data, including platelet count, white blood cell (WBC) count, and hematocrit (HCT). These parameters were measured using an automated hematology analyzer, with hematocrit additionally verified by microcentrifugation. The results show that these parameters remained relatively stable over the 4-hour loop study, despite potential sublethal damage to some cells.

Platelet count (103/mm3)

|  | BrioVAD | HM3 |
| --- | --- | --- |
| base | 154.5 | 156.818 |
| 0H | 139.9 | 145.727 |
| 1H | 143.3 | 140.636 |
| 2H | 143.1 | 137.909 |
| 3H | 142 | 138.636 |
| 4H | 143.3 | 142.364 |

**Figure S3**. Platelet count.

WBC count (103/mm3)

|  | BrioVAD | HM3 |
| --- | --- | --- |
| base | 4.47 | 4.56364 |
| 0H | 4.37 | 4.43636 |
| 1H | 4.43 | 4.5 |
| 2H | 4.51 | 4.52727 |
| 3H | 4.56 | 4.72727 |
| 4H | 4.66 | 5.00909 |

**Figure S4**. WBC count.

HCT(%)

|  | BrioVAD | HM3 |
| --- | --- | --- |
| base | 33.1 | 33.0909 |
| 0H | 32.6 | 32.8182 |
| 1H | 32.8 | 32.6364 |
| 2H | 32.8 | 32.7273 |
| 3H | 32.9 | 32.7273 |
| 4H | 33 | 32.9091 |

**Figure S5**. HCT.

## Flow Cytometry

**Figure S6** shows the analysis strategy for platelet activation and receptor shedding. Platelets were first identified by gating the CD41a⁺/SSC^low population. Activated platelets were defined as CD62P⁺ or PAC-1⁺ events within the CD41a⁺ population. Receptor shedding was assessed by measuring the expression levels of GPIbα, GPVI, and GPIIb/IIIa within the CD41a⁺ population. Representative density plots are shown from a blood sample in the BrioVAD loop at hour 4. The flow cytometry gating strategy for neutrophils has been described in detail in the main manuscript and our previous publications [22].

**Figure S6**. The flow cytometry gating strategy for platelet activation and receptor shedding.
