# Supplementary material for: Device‐Induced Blood Damage in Pump‐Assisted Circulation: A Comparative Study of HeartMate III and BrioVAD Pumps
Source: Artif Organs. 2026 May 12;50(9):1295–304. doi: 10.1111/aor.70166 (PMC13525217; doi:10.1111/aor.70166)
Supplement: Supplementary file 2 [file AOR-50-1295-s002.docx]

Supplemental Table 1. List of monoclonal antibodies used in this study.

| **Specificity** | **mAb clone** | **Ig Isotype** | **Fluorescent label** | **Manufacturer** |
| --- | --- | --- | --- | --- |
| Pac-1 | Pac-1 | Mouse IgM, ĸ | FITC | BD biosciences |
| P-selectin | AK4 | Mouse IgG1, ĸ | PE | BioLegend |
| GP Ibα | HIP1 | Mouse IgG1, ĸ | FITC | BD biosciences |
| GP VI | HY101 | Mouse IgG1, ĸ | eFluro660 | ThermoFisher |
| GP IIb/IIIa | 2A9/6 | Mouse IgG2a, ĸ | PerCP-CY5.5 | BioLegend |
| CD41a | HIP8 | Mouse IgG1, ĸ | APC | BD biosciences |
| CD66b | G10F5 | Mouse IgM, ĸ | BD Horizon™ BV450 | BD biosciences |
| CD62L | DREG-56 | Mouse IgG1, ĸ | FITC | BD biosciences |
| CD11b | CBRM1/5 | Mouse IgG1, ĸ | PE | BioLegend |
| CD11a | TS2/4 | Mouse IgG1, ĸ | PerCP | BioLegend |
| Isotype | G155-228 | Mouse IgM, ĸ | FITC | BD biosciences |
| Isotype | MOPC-173 | Mouse IgG2a, ĸ | PerCP-CY5.5 | BioLegend |
| Isotype | MOPC-21 | Mouse IgG1, ĸ | FITC, PE, APC | BD biosciences |
| Isotype | MOPC-21 | Mouse IgG1, ĸ | PerCP | BioLegend |

Supplemental Table 2. Baseline values for all the biomarker measurements.

| **Biomarker** | **Baseline value** | |
| --- | --- | --- |
|  | HM3 | BrioVAD |
| Pac-1 (%) | 0.62 ± 0.09 | 0.61 ± 0.10 |
| P-selectin (%) | 1.07 ± 0.10 | 1.05 ± 0.09 |
| GPIbα (MFI) | 359.73 ± 15.18 | 363.00 ± 16.73 |
| GP VI (MFI) | 166.64 ± 9.61 | 166.67 ± 11.17 |
| GP IIb/IIIa (MFI) | 190.00 ± 9.20 | 185.11 ± 11.76 |
| Annexin V (%) | 1.94 ± 0.17 | 1.83 ± 0.19 |
| PMPs (%) | 1.70 ± 0.16 | 1.58 ± 0.16 |
| TMRE (MFI) | 242.50 ± 17.70 | 239.60 ± 17.82 |
| CD62L (MFI) | 3611.80 ± 133.47 | 3641.67 ± 149.13 |
| CD11b (%) | 1.88 ± 0.57 | 1.77 ± 0.70 |
| CD11a (MFI) | 2831.78 ± 113.77 | 2867.00 ± 122.67 |
| CD66b (MFI) | 3388.73 ± 208.99 | 3395.90 ± 220.45 |
| Phagocytosis (%) | 3.31 ± 0.78 | 3.13 ± 0.79 |
| HMWM VWF (%) | 53.56 ± 1.71 | 53.15 ± 2.16 |
